# Ankle Torque Estimation With Motor Unit Discharges in Residual Muscles Following Lower-Limb Amputation

**DOI:** 10.1109/TNSRE.2023.3336543

**Published:** 2023-12-08

**Authors:** Noah Rubin, Robert Hinson, Katherine Saul, Xiaogang Hu, He Huang

**Affiliations:** Department of Biomedical Engineering, The University of North Carolina at Chapel Hill, Chapel Hill, NC 27599 USA; Joint Department of Biomedical Engineering, NC State University, Raleigh, NC 27695 USA; Rehabilitation Medicine Department, Clinical Center, National Institutes of Health, Bethesda, MD 20892 USA; UNC-Chapel Hill School of Medicine, Chapel Hill, NC 27599 USA; Department of Mechanical and Aerospace Engineering, NCSU, Raleigh, NC 27695 USA; Penn State College of Human Health & Development, University Park, PA 16802 USA; Joint Department of Biomedical Engineering, The University of North Carolina at Chapel Hill, Chapel Hill, NC 27599 USA; Joint Department of Biomedical Engineering, NC State University, Raleigh, NC 27695 USA

**Keywords:** Amputation, EMG, motor unit, neural-machine interface, prosthesis control

## Abstract

There has been increased interest in using residual muscle activity for neural control of powered lower-limb prostheses. However, only surface electromyography (EMG)-based decoders have been investigated. This study aims to investigate the potential of using motor unit (MU)-based decoding methods as an alternative to EMG-based intent recognition for ankle torque estimation. Eight people without amputation (NON) and seven people with amputation (AMP) participated in the experiments. Subjects conducted isometric dorsi- and plantarflexion with their intact limb by tracing desired muscle activity of the tibialis anterior (TA) and gastrocnemius (GA) while ankle torque was recorded. To match phantom limb and intact limb activity, AMP mirrored muscle activation with their residual TA and GA. We compared neuromuscular decoders (linear regression) for ankle joint torque estimation based on 1) EMG amplitude (aEMG), 2) Mu firing frequencies representing neural drive (ND), and 3) MU firings convolved with modeled twitch forces (MUDrive). In addition, sensitivity analysis and dimensionality reduction of optimization were performed on the MUDrive method to further improve its practical value. Our results suggest MUDrive significantly outperforms (lower root-mean-square error) EMG and ND methods in muscles of NON, as well as both intact and residual muscles of AMP. Reducing the number of optimized MUDrive parameters degraded performance. Even so, optimization computational time was reduced and MUDrive still outperformed aEMG. Our outcomes indicate integrating MU discharges with modeled biomechanical outputs may provide a more accurate torque control signal than direct EMG control of assistive, lower-limb devices, such as exoskeletons and powered prostheses.

## I. Introduction

Recent advances in powered lower-limb prostheses have shown great potential to improve the mobility of individuals with lower-limb amputations. Although most commercialized lower-limb prostheses have been passive, the last decade has brought powered devices to the market to improve mobility of prosthesis users. Powered lower-limb prostheses are typically operated autonomously to assist various biomechanically well-defined, cyclic locomotion modes, such as level walking, stair ascent/descent, etc. [1]. Still, it is difficult to pre-program autonomous controllers for complex or unique biomechanics necessary in many activities of daily living such as sit-to-stand or squatting, as well as recreational activities (e.g., free-style dancing, rock climbing etc.). In these scenarios, dynamic postural adjustments occur, and autonomous controllers alone do not account for volitional desire of users to appropriately modify limb dynamics.

To address this challenge, designing a neural-machine interface has become a popular approach to directly integrate the user’s intent in driving control of assistive and rehabilitative devices [2], [3], [4]. Among these techniques, surface electromyography (EMG) is a popular choice for neural prosthesis control because it provides a noninvasive and straightforward setup, with neural information from residual muscles after amputation available to allow identifying motor intent of the missing limb [5]. Nevertheless, myoelectric control has been mainly used for upper-limb prostheses in clinics. It has only emerged in recent years as a potential solution for powered lower-limb prosthesis control.

There are two major EMG-based neural interface designs for powered lower-limb prostheses [6]. First, studies have decoded EMG to drive prosthesis control for seamless transitions between locomotion modes [7], [8]. EMG signals of residual muscles, sometimes combined with other intrinsic motion and force sensors, are sent to a pattern classifier to predict the user’s locomotion mode in the next step, which then switched prosthesis control mode accordingly. Although promising, this interface is only useful during well-defined locomotion tasks (e.g., level walking and stair ascent/descent). It is still incapable of enabling users to perform unpredictable tasks. Another existing design of an EMG-based neural interface used antagonistic muscle activity to modify prosthesis joint mechanics (such as torque or impedance) directly and continuously [9], [10]. This more directly mimics ways in which a biological limb generates motion. For example, in one application of this strategy, the magnitude of the residual *tibialis anterior* (TA) and *gastrocnemius* (GA), i.e., antagonistic muscles for ankle dorsi- and plantarflexion control, were used to produce ankle torque to drive the motion of a prosthesis ankle [10]. This design gives the prosthesis users full freedom to operate the powered prosthesis joint based on their intent. It has been applied to cyclic and walking tasks, dynamic postural control under anticipatory postural adjustment to perturbations, and even rock climbing [11].

When using direct continuous myoelectric control for prosthetic legs, muscle activity is typically quantified as the magnitude of the EMG signals (aEMG), which is a gross estimation of neural motor control commands. The EMG signal is a convolution of measurable motor units’ action potentials (MUAPs) and respective firing times [12]. In order to accurately decode neuromuscular control and estimate the user’s intended joint torque, using MUAPs and spike trains may be more precise than the magnitude of EMG signals. When using EMG magnitude to represent neural drive, signal loss occurs from cancellation of overlapping MUAPs [13]. Alternatively, modern techniques in electrode design and source separation algorithms have decomposed EMG into spike trains [14], [15]. Descending neural drive is directly reflected in populational MU firing rates, as human motor control commands that lead to muscle force, joint torque, and joint motion is encoded by motor units’ recruitment and firing rates [16]. By using binary firings, signal loss from MUAP cancellation along with noise interference are also mitigated. Indeed, use of MU firing rates to decode motor intent, as well as combining firings with modeled twitch forces [17] has accumulated and shown promise to be more accurate (in terms of muscle force and joint kinematics) than using conventional EMG amplitude [18], [19]. Still, most reports investigating MU-based motor decoding have been conducted in the upper-limb [17], [20], [21].

While studies on myoelectric prostheses in the lower-limb continue to grow, little work has investigated differences in accuracy of EMG-based versus MU-based continuous motor intent decoding in the lower-limb in either intact (Int) muscle or residual (Res) muscle following amputation. There are fundamental differences between the upper- and lower-limb in control strategy, anatomy, muscle architecture [22], MU sizes, and coding of activation through increases in MU recruitment versus firing rates [23], all of which impact EMG generation [24] – it is unclear how decoder performance between these methods may be impacted. To address this knowledge gap, we asked people with amputation below the knee (AMP) and people without amputation (NON) to modulate activation of intact and residual ankle dorsi- and plantarflexors. In analysis, we compared the accuracy of three decoders in estimating recorded ankle torque based on 1) EMG amplitude (aEMG), 2) MU firing frequencies representing neural drive (ND), and 3) MU firings convolved with modeled twitch forces (MUDrive). Based on advantages in representing neural drive with MU firing rates, we hypothesized MU-based decoders would outperform EMG in both intact and residual muscles.

## II. Methods

### A. Subject Recruitment and Testing Days

Eight AMP with residual limb lengths at least 10 cm and capability to walk across low-level environmental barriers such as curbs, stairs, or uneven surfaces and eight NON were recruited for this study. Table I provides detailed subject characteristics of AMP (for NON, 7 male, 1 female, aged 29.4±3.25 years, 177±6.37cm tall, with mass of 76.4±9.41kg were recruited). AMP participated in two different sessions spaced at least two days apart. The first session included time to search for appropriate placement of sensors on residual muscles, familiarization with the user interface for visual feedback, and practice controlling muscle activation. The full experimental protocol was conducted in the second session. NON underwent the entire study in one session. All subjects provided written informed consent for protocols approved by the North Carolina State University Institutional Review Board (Protocol #24436). One AMP (73-year-old, male) was unable to track visual feedback both early on and after practice and was excluded. Note AMP4 had a partial foot amputation on their contralateral limb. This subject had lower max torque output relative to other AMP but could maintain reasonable modulation and control of intact ankle torque and muscle activation on both limbs, so they were retained in analysis.

### B. Experimental Protocol

#### 1) Setup:

**Fig. 1.**
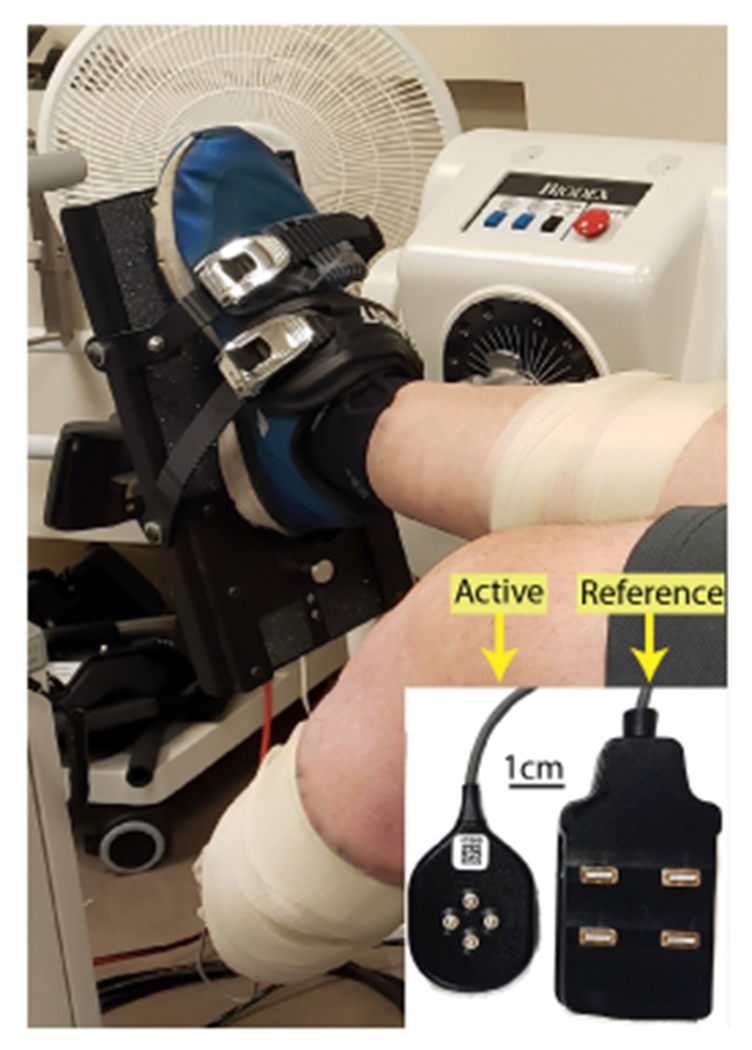
Experimental Setup. AMP had their intact limb fixed to an ankle attachment such that all muscle activation was isometric. The inset shows the active and reference electrodes placed on the muscle and a nearby bony landmark, respectively. NON had each limb tested in the ankle attachment in the same configuration.

EMG was recorded using four 4-pin surface electrodes (Galileo, Delsys, Inc., Natick, MA). Electrodes were placed on the TA and lateral GA of each limb. For AMP, their intact limb was fixed to an ankle attachment in all tasks, with the ankle parallel to the axis of rotation of a dynamometer (System 4 Pro, Biodex, Shirley, NY, Figure 1). The intact ankle was at a neutral angle, and the knee had 5 degrees of flexion to permit greater plantarflexion torque [25]. The residual limb rested off the chair to prevent extraneous muscle activity and minimize artifacts in recorded EMG. For NON, the targeted limb was set up in the same manner as the intact limb of AMP, while the non-targeted limb was bent resting off the chair. Torque data were recorded at 100 Hz to an external data acquisition system (MATLAB 2021b, NI USB-6003). EMG data were recorded at a sample rate of 2,222 Hz (EMGWorks, version 4.7.9) and bandpass filtered from 20-450 Hz [26]. Data were synchronized with the EMG device as primary and the NI DAQ as secondary (Trigger Module, Delsys, Inc.).

#### 2) Muscle Activation Calibration:

Before experimental trials, subjects conducted a maximum voluntary contraction (MVC) for 3-sec for each of the 4 muscles (random order) two times, with a minimum of 1-min of rest between each MVC. The MVC was calculated based on the largest 0.5-sec window of the root-mean-square (RMS) of a random channel of the EMG sensor. For all visual feedback thereafter, muscle activation was provided as a percentage of MVC.

#### 3) Experimental Task: Profile Tracking of Muscle Activation:

**Fig. 2.**
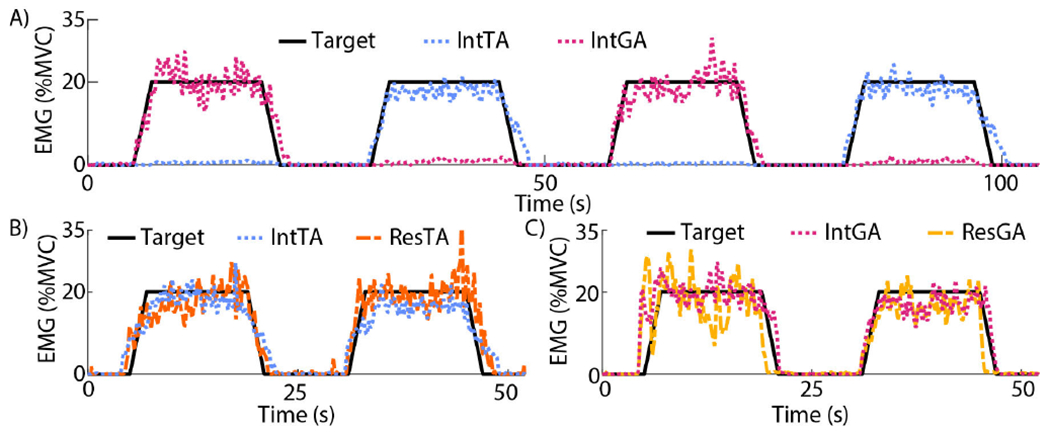
Representative experimental tasks by AMP2. A) Intact limb. Subjects were provided a target profile (solid black) they matched by alternately controlling muscle activation (as %MVC) of their TA and GA. EMG data from the intact TA (dotted blue) and GA (dotted red) muscles were used to estimate dorsi- and plantarflexion torque of their ankle, respectively. B-C) Residual limb. Subjects were provided feedback of bilateral muscles in two different trials, and simultaneously mirrored activation of the intact muscle with the residual B) TA and C) GA muscles. EMG data from residual TA (dashed orange) and GA (dashed yellow) muscles were used to predict ankle torque produced by their intact limb.

To estimate torque at different levels of activation, subjects traced targeted %MVC levels in profiles shaped like trapezoids (two or four depending on the limb, Figure 2). Each trapezoid had a 10 %MVC/sec ramp up to the targeted MVC level, followed by a 12-sec steady-state period, and a 10 %MVC/sec ramp down. The trial began with a 5-sec quiescent period, and a 10-sec rest period was allotted between each trapezoid within a trial. At least 1-min of rest was provided between each trial. For each task, subjects targeted 20 and 35 %MVC for two trials each. Prior to the experimental trials for dorsi- and plantarflexion in each limb, subjects practiced the 20 %MVC profile two to four times until they were able to adapt to visual feedback and reasonably trace the target. Subjects did not practice the 35 %MVC target to prevent fatigue. These target levels were chosen after pilot testing targeting 10, 20, 30, and 40 %MVC at steady state. Minimal activation was observed at 10 %MVC at steady-state, while 40 %MVC was difficult for subjects to maintain. Each limb was tested in random order as described below.

#### 4) Intact Limb Task:

When testing the intact limb for AMP, and each limb for NON, a unilateral task was conducted. The respective TA and GA muscle activations of the targeted limb were provided to subjects using visual feedback. The target trial consisted of four trapezoids, where subjects sequentially alternated plantar- and dorsiflexion on each trapezoid (random direction for the first trapezoid, Figure 2A).

#### 5) Residual Limb Tasks:

When testing the residual limb for AMP, there is no joint available to record a ground truth of desired ankle torque for the phantom limb. A mirrored, bilateral task was conducted as a reasonable approximation of motor intent for a phantom limb of a person with unilateral amputation [27]. The respective bilateral TA or GA muscle activations for the non-amputated (intact) and residual limbs were provided to subjects. Tasks for the TA and GA activation were separated to allow for only two channels of feedback at a given time and prevent confusion for subjects. In each trial, subjects conducted bilateral, mirrored activation of the targeted TA (Figure 2B) or GA (Figure 2C) muscles in two repeated trapezoids. In later processing for these tasks, EMG of residual muscle was used to estimate recorded torque on the non-amputated limb.

### C. Data Processing

#### 1) Postprocessing:

Torque data from each trial were low-pass filtered (2 Hz cutoff [28] because of the slow task, 4th order Butterworth) after DC offset removal (mean of the first 1-sec of data) and averaged in a moving window (0.5-sec, 0.1-sec step) to downsample and serve as a reference to estimate. MUs were decomposed from EMG data with Neuromap (Delsys, Inc.), built off a maximum a posteriori classifier that updates a template library of MU action potentials to minimize the residual between a synthesized and measured EMG signal [14]. This technique has undergone prior independent experimental validation with a two-source test [29] and rigorous simulation [30]. In this study, decomposed MUs were validated with a 2-step spike-triggered averaging (STA) procedure. Briefly, an 80-ms window of the filtered EMG data at every time of a MU firing was stacked and averaged across all firings to compute an STA template of the MUAP. If the normalized cross-correlation of the STA template and decomposed MUAP had a coefficient of determination (R^2^) greater than 0.6, the decomposition was deemed accurate [31]. Additionally, the STA was computed in a moving window within the trial (8-sec window, 4-sec step). To confirm stability of the decomposition over time, MUs were retained if the coefficient of variation (CV) of the peak-to-peak amplitude of the MUAP across the segmented STAs was below 0.5 [31].

#### 2) Model Training and Testing:

For residual muscle of AMP, each model could only be fitted to a single muscle due to independence of residual TA and GA tasks as noted above. Because of this, independent models were built for non-amputated TA and GA muscles to estimate dorsi- and plantarflexion torque, respectively. For trials targeting non-amputated muscles, data of the four trapezoids repeated in each trial were reorganized into two sets of two trapezoids, each consisting of either repeated targeted TA or GA activation, to align with data of tasks for residual muscles. For each method described below, models for each muscle were trained and tested on these data in a two-fold cross-validation, split in half between the two trapezoids. In all methods prior to model training and testing, the input was cross-correlated and aligned with reference torque to account for electromechanical delay in torque generation for intact limbs and human error in mirrored residual limb tasks.

#### 3) EMG & Neural Drive Model Training:

**Fig. 3.**
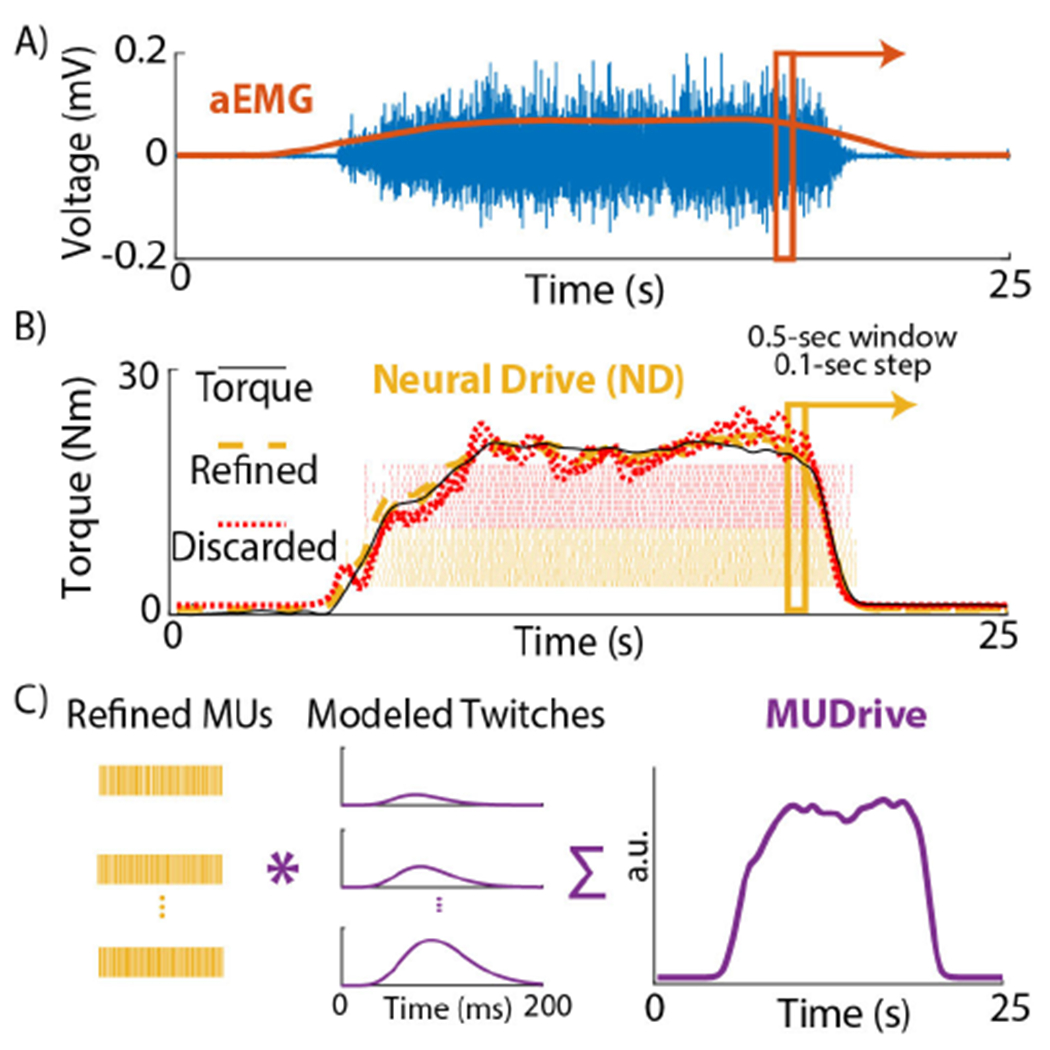
Torque Estimation Methods. A) EMG amplitude (aEMG). A moving window computed the root-mean-square (RMS, orange) of the EMG (blue) channel used for visual feedback to control muscle activation. RMS data were linearly regressed to recorded ankle torque. B) Neural Drive (ND). The same moving window computed firing rates of each decomposed MU based on discharge times. Each MU firing rate was linearly regressed to recorded torque. MUs that did not correlate with torque were discarded (dotted red), and refined MU firing rates (dashed yellow) estimated torque in multiple linear regression. C) MUDrive. Refined MU discharge times were convolved with modeled twitch forces and summed to compute MUDrive (purple), which estimated torque via linear regression.

For the EMG amplitude (aEMG) method (Figure 3A), the RMS of the EMG signal (0.5-sec window, 0.1-second step) was calculated from the same channel used in visual feedback and normalized to MVC to mitigate effects of MUAP cancellation [13]. Thereafter, a Kalman filter smoothed the signal. The normalized, smoothed aEMG signal was linearly regressed to recorded torque. For the neural drive (ND) method (Figure 3B), firing rates of each valid decomposed MU were computed (using the same window and Kalman filter), and linearly regressed to torque. The R^2^ was calculated between estimated and measured torque, and the 10 MUs with the highest R^2^ values were retained to refine estimation (all were retained if less than 10 MUs were decomposed) [28]. The refined smoothed firing rate *F R* for each *i*th MU was fit to a multiple linear regression model to estimate torque *τ* at each time *t*:

(1) $$\tau \left(t\right)=\sum_{i}a_{i}F\;R_{i}\left(t\right)+b$$

where *a_i_* and *b* are fitted coefficients and the intercept.

#### 4) MUDrive Model Training:

The same refined MUs used in the ND method were kept for the MUDrive method. The MUDrive method is derived from a simulation model that estimates a modeled force twitch profile for a given MU [32]. Twitch profiles of refined MUs were convolved with their respective decomposed spike trains and summed to compute a MUDrive signal over time, representing modeled force (Figure 3C). Twitches of larger MUs tend to have longer rise times and half-relaxation times, and larger peak force [33]. As done in [17], a genetic algorithm optimized MUDrive model performance to determine the range of peak twitch amplitude (*P*), rise times (*T_r_*), and half-relaxation times (*T_hr_*) for the MU pool used in the model for each trial (see Supplementary Material for details on each variable). In this study, based on reference data of the vastus lateralis [32] (to our knowledge no reference data on the muscles studied was available), the constrained physiologic realizable ranges for both the TA and GA to determine *P_max_*, *T_r_min__*, *T_r__max_*, *T_hr_min__*, and *T_hr_max__* were conservatively selected to be 1.1-150 (arb. unit), 30-110 ms, 150-240 ms, 15-60 ms, and 110-140 ms, respectively (with *P_min_* predefined at 1). These three parameters for a given MU were computed as a linear rescaling to the range for the MU pool based on its recruitment threshold (RT), according to the size principle [34]. The RT for each MU was defined as the %MVC of the EMG signal amplitude in a 200 ms window centered at the time of the first firing when the MU firing rate was greater than 5 Hz. The same moving window and Kalman filter were applied to the MUDrive signal, and the smoothed output was linearly regressed to the recorded torque. This method has been implemented in real time decoding motor intent for AMP in the upper-limb [17] (see Supplementary Material for details).

#### 5) Evaluation & Comparing Model Performance:

The root-mean-square error (RMSE) between estimated and measured torque in model testing data for each cross-fold was averaged. No model training data was used for evaluation. For each muscle and subject, there were two responses of RMSE at each targeted %MVC level. Each muscle had an independent statistical model (RMSE as the response). A mixed effect model with fixed effects of method (aEMG, ND, MUDrive) and targeted %MVC level (20, 35) was used (subjects as random intercept). Data were tested for normality to determine use of a parametric or nonparametric model (α=0.05). If significant differences were detected, multiple pairwise comparisons were conducted using Student’s paired t-tests and a Bonferonni correction.

#### 6) Refinement of MUDrive Optimization:

To further investigate MUDrive as an alternative to EMG-based decoding, sensitivity analyses were conducted on each parameter to determine which parameters have the largest effect on variations in model performance. Following this, informed refinement of model optimization was conducted to test if optimization can be simplified without sacrificing performance. For the sensitivity analysis, in each trial, 4 of 5 parameters were fixed to the optimized value used in initial performance evaluation. The remaining parameter was perturbed randomly in a uniform distribution across the constrained physiologic bounds (see MUDrive Model Training) in a Monte Carlo simulation [35]. Simulations were conducted until the standard deviation (SD) of RMSE across all simulations converged, such that the final 50 iterations did not change the SD by more than a tolerance of 10^−4^, and a minimum of 1,000 iterations were conducted for each simulation. For each muscle studied, parameter sensitivities were ranked based on converged SD of RMSE (higher SD indicates higher sensitivity). After ranking parameter sensitivities, refinement of optimization was conducted. As a baseline, zero parameters were optimized, and all were fixed to the midpoint of constrained bounds. Following this, an increasing number of parameters were optimized, ordered by most to least sensitive. Model performance and optimization time were computed based on the number of parameters used in optimization. Paired t-tests to outcomes with full parameter optimization were conducted to evaluate differences in performance with model refinement [36]. Simulations were processed on a desktop (Intel i7-9700, 24 GB RAM).

## III. Results

The number of MUs decomposed across all trials ranged from a minimum of 6 (Intact GA) up to 45 (GA of NON). The mean and standard deviation across subjects were 18.8±5.6, 22.1±7.5, and 21.7±7.9 MUs for the residual and intact TA and TA of NON, respectively. For the GA, 20.0±7.0, 18.4±8.8, and 22.6±9.6 MUs were decomposed in the residual and intact GA and GA of NON, respectively.

**Fig. 4.**
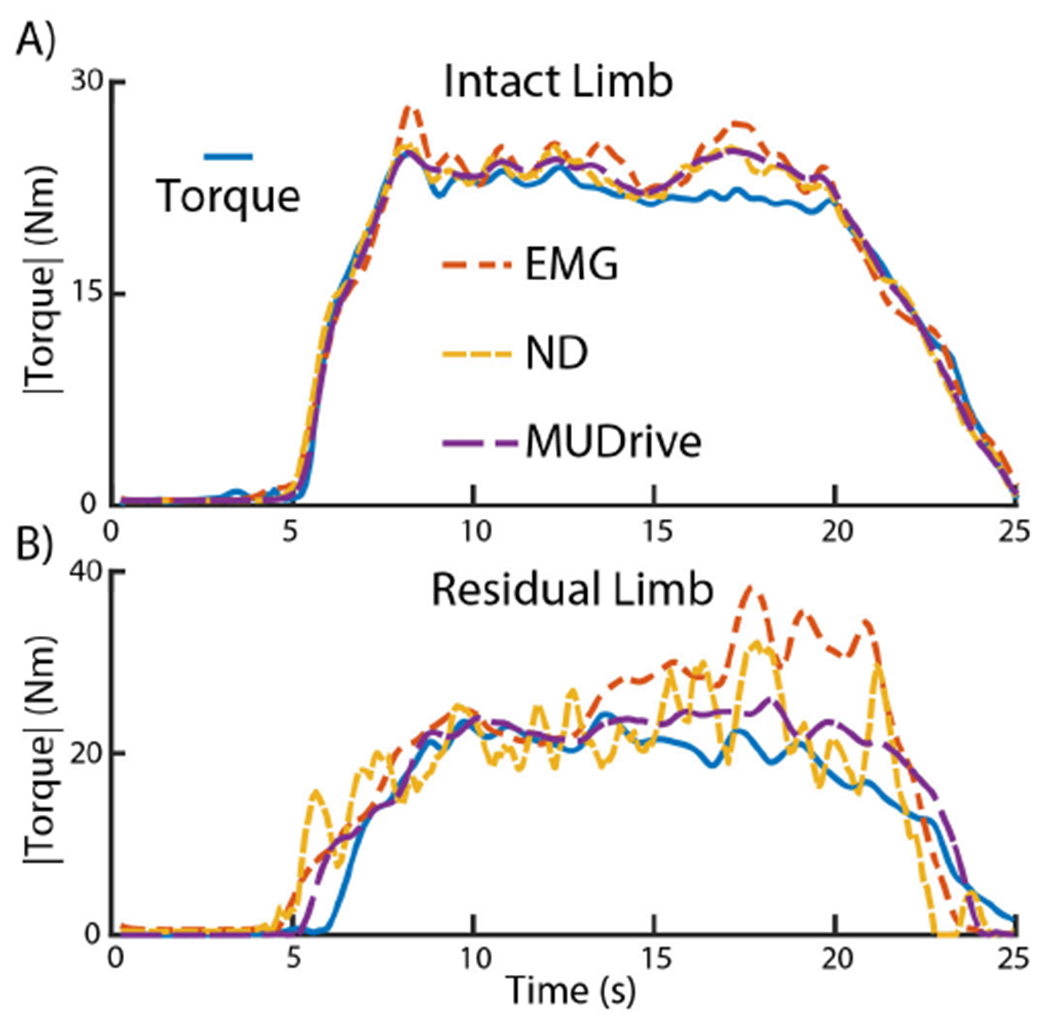
Representative model performances in ankle torque estimation. Three methods (aEMG, ND, and MUDrive as orange, yellow, and purple dashed lines respectively) were used to estimate recorded ankle torque (solid blue). Data shown is from the TA of AMP2 targeting 35 %MVC for the A) Intact and B) Residual limb tasks. The MUDrive method exhibited smoother estimations, with less drift present in the residual limb task.

In the intact limb task (Figure 4A), all methods closely follow the torque profile at low levels and during changes in torque output. During the steady-state period, however, aEMG exhibits greater oscillations away from the torque reference compared to MU-based estimates. In the residual limb (Figure 4B), estimates do not follow torque as closely as the intact limb, but all methods maintain reasonable performance. During the early quiescent period, all methods matched torque with zero outputs. Even though signals were cross-correlated and shifted, there were delays in estimates during changes in torque due to human error in maintaining exactly mirrored muscle activation. During both changes in torque and steady-state, the aEMG and ND methods displayed more oscillation compared to the MUDrive method. During the second half of the steady-state period, while the torque level did not change, the aEMG estimate drifted by more than 5 Nm, compared to the MUDrive method, which maintained a similar torque estimate throughout the steady-state period.

**Fig. 5.**
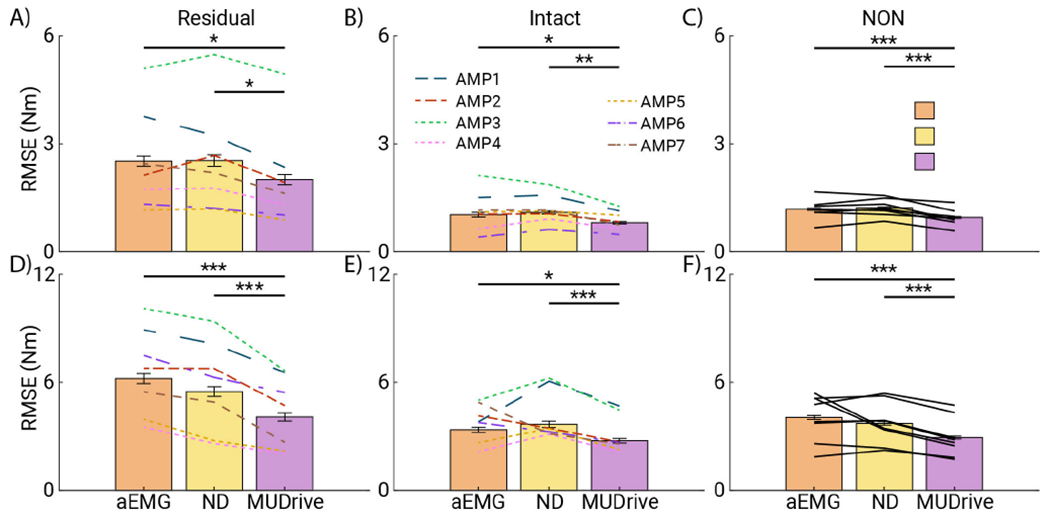
Summary of performance for each method in each muscle. Panels in the top row show data from the TA of the A) residual limb, B) intact limb, and C) limbs of people without amputation (NON). The bottom row (D-F) shows data from the GA muscles for the respective limbs corresponding to the top row. Each bar displays the mean and standard error for each method across all trials in both targeted MVC levels (from left to right, aEMG, ND, and MUDrive, respectively). Line plots show averages for each subject (*p<0.05, **p<0.01, ***p<0.001).

All data of performance (RMSE) for each method and targeted %MVC were normally distributed. Significant differences in method and %MVC level were observed in all conditions, but no significant interaction effect between method and targeted %MVC level was detected (See Supplementary Material for details). In post-hoc analyses, the targeted 35 %MVC level resulted in significantly higher error compared to the 20 %MVC level. Because no interaction was observed, results across %MVC levels were combined to summarize data for each method as the primary research question. Figure 5 displays model performances for each method in each muscle across both %MVC levels. As observed in the representative example, performance in the residual muscle was consistently worse than intact muscle. In the residual TA, intact TA, and TA of people without amputation (NON), aEMG and ND performed similarly (Cohen’s d = 0.026, 0.12, and 0.13, respectively), while MUDrive significantly outperformed both methods (in each respective limb, Cohen’s d = 1.14, 1.16, and 1.36 comparing to ND, and 0.92, 0.44, and 0.71 comparing to aEMG). In the residual GA, both ND and MUDrive significantly outperformed aEMG. Although ND was worse than aEMG in the intact GA, MUDrive significantly outperformed aEMG in this muscle, and both ND and MUDrive significantly outperformed aEMG in the GA of NON, with MUDrive again resulting in the highest performance. No strong trend was found between the number of MUs extracted and MUDrive performance across conditions (all *R*^2^ <0.1 except intact GA *R*^2^=0.32, linear fit).

When comparing performance among AMP, overall performance varied (solid and dashed line plots). However, except for AMP2 and AMP3 in the residual TA (Figure 5A), along with AMP1 and AMP3 in the intact GA (Figure 5E), most AMP displayed similar trends in differences in performance between methods. Notably, although aEMG and ND did not have significant performance differences in any muscle, MUDrive significantly outperformed aEMG and ND in all muscles (Figure 5).

### A. MUDrive Model Sensitivity Analysis & Model Refinement

**Fig. 6.**
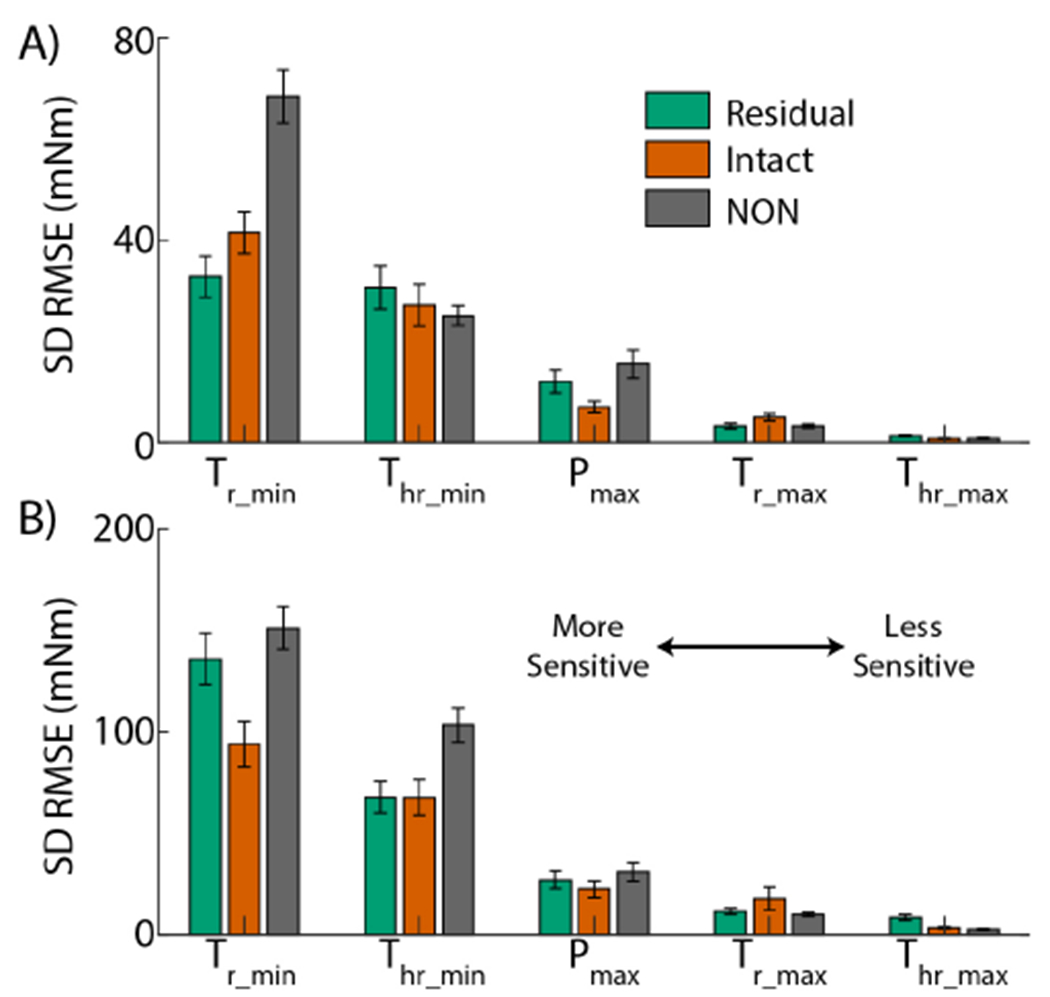
MUDrive Sensitivity Analysis. Each bar is the mean and standard error of the standard deviation (SD) of model performance across all iterations of a Monte Carlo simulation perturbing each parameter for the A) TA and B) GA. In both muscles, the residual (green) and intact (red) limbs and limbs of NON (dark gray) resulted in the same ranking order of parameter sensitivity.

In the TA (Figure 6A), NON had higher sensitivity than residual and intact limbs for *T*_r_min__, but lower sensitivity to *T_hr_*_*min*_. The intact limb had the lowest sensitivity to *P*_*max*_, and the highest sensitivity to *T_r_max__*. *T*_*hr_max_*_ displayed little sensitivity in all limbs. In the GA (Figure 6B), the intact limb was least sensitive in *T_r_min__*, while NON had the most sensitivity to *T_hr_min__*. All limbs had similar sensitivity to *P*_*max*_. Intact and residual limbs had higher sensitivity than NON for *T_r_max__* and *T_hr_max__*, respectively. Although there was large variation in sensitivity between limbs for a given parameter, all three limbs and both muscles exhibited the same trend of parameter sensitivities, with *T_r_min__*, *T_hr_min__*, *P_max_*, *T_r_max__*, and *T_hr_max__* ranked from most to least sensitive, respectively.

Based on these rankings, each parameter was sequentially included (from most to least sensitive) in optimizations to observe the impact of model refinement on model performance and optimization time. The number of parameters needed to match the performance of a full model varied between limbs and muscles (Table II). For NON, only three parameters were needed to reach comparable performance to a fully optimized model in both the TA and GA. For AMP, two and three parameters needed optimization in the intact TA and GA, respectively. For the residual limb, four parameters were needed in the TA, and no refinement matched full performance in the GA. While performance significantly worsened with model refinement, remarkably, performance without any optimization still produced better outcomes than the aEMG method in all limbs for both the TA and GA (Figure 5).

**Fig. 7.**
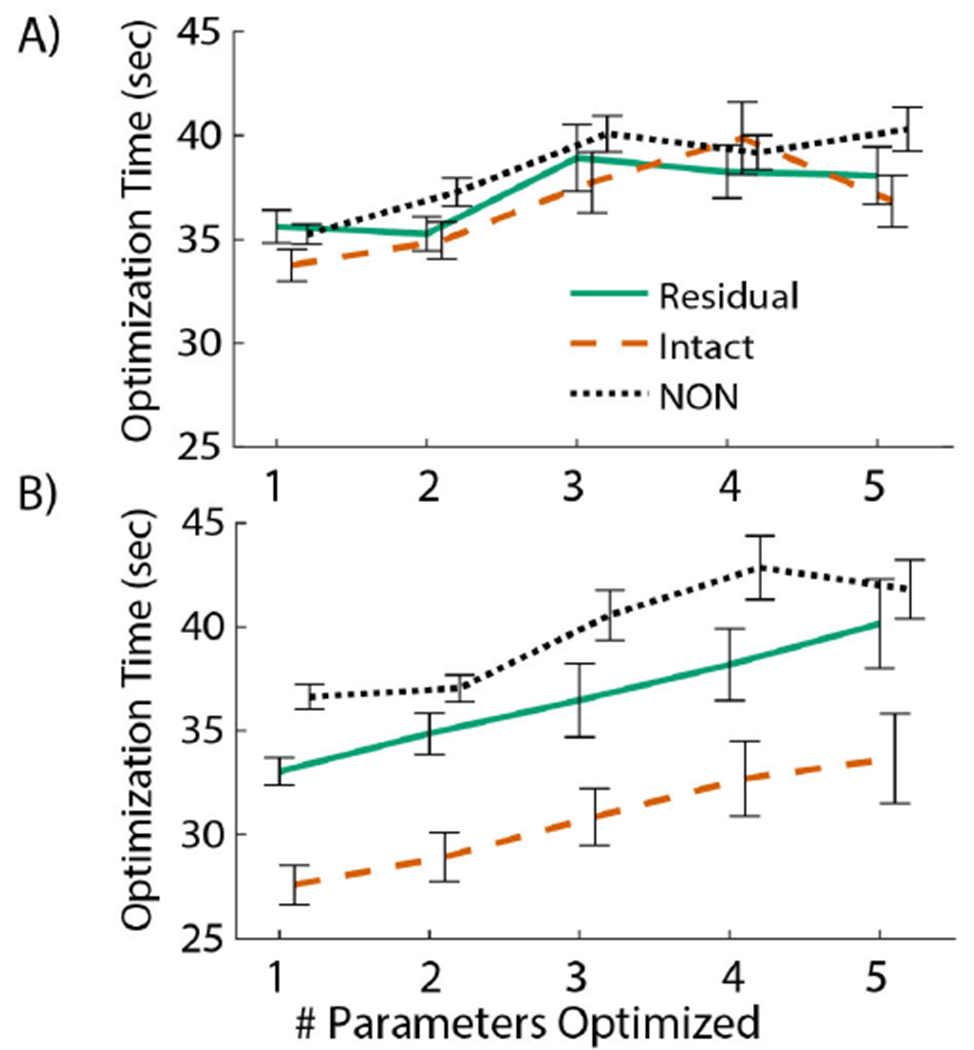
Change in optimization time of the MUDrive model in each trial relative to full parameter optimization for the A) TA and B) GA. Line plots indicate mean and standard error across all trials for the residual (solid green) and intact (dashed orange) muscles and muscles from NON (dotted black).

For percent changes in optimization time (Figure 7), similar trends were observed across limbs and muscles. When optimizing all parameters in each trial (approximately 26-29 sec of training data depending on the targeted %MVC level), optimization time generally ranged from 30-45 sec. Apart from optimizing four parameters for the intact TA or GA of NON, reducing parameters resulted in similar or faster time to convergence. In both the TA (7A) and GA (7B), optimization using only one parameter was faster compared to a fully optimized MUDrive model (Figure 5).

## IV. Discussion

This study evaluated continuous prediction accuracy of isometric ankle torque using conventional EMG amplitude (aEMG), MU firings (ND), and MU firings convolved with a twitch force model (MUDrive) of intact and residual muscles. Prior work exploring application of these methods to lower-limb amputations has been very limited. Our results suggest MUDrive outperforms EMG and ND methods in muscles of people without amputation, as well as both intact and residual muscles of people with amputation below the knee. Reducing the number of optimized parameters in the twitch force model compromised performance but consistently maintained better outcomes than EMG while improving optimization time. Together, our findings indicate a more physiologic-inspired model of torque estimation may readily improve the accuracy of future myoelectric control of powered ankle prostheses.

### A. Ankle Torque Estimation Accuracy

We observed significantly better performance of the MUDrive method compared to both the aEMG and ND methods in all muscles tested. With the ND method, differences among individual MUs were accounted for with tuned constant coefficients determined in multiple linear regression. Instead, MUDrive used gain factors adaptive to the instantaneous firing rate. Additionally, the ND method directly regressed MU firing rates to torque. The MUDrive method convolved spikes with unique optimized twitch forces that acted as second-order damped systems [37]. The greater complexity of the MUDrive model provided a larger range of outputs (better resolution) for the same firing rate, so discharges combined with modeled twitch forces into the MUDrive signal produced smoother signal estimates compared to EMG (also observed in [17]) and ND signals, significantly improving prediction accuracy.

Beyond smoothing signal estimates with greater model complexity, the MU-based methods may have improved the quality of the input signal to the model. As described earlier, decomposition recovers signal loss from overlap of MUAPs in the EMG. We conducted normalization of the EMG amplitude to mitigate this effect in the conventional method, but any cancellation that does occur cannot be entirely counteracted with this procedure [13]. Instead, using MU firing rates based on binary spike trains was robust to any MUAP overlap or noise that may have occurred. In addition to signal loss from cancellation, EMG may have been contaminated with some degree of crosstalk. For plantarflexion estimation, we placed electrodes on the lateral GA. The differential spatial filter in recorded EMG reduces crosstalk from the soleus and medial GA, but does not entirely remove this interference [38]. While these muscle heads produce mechanically synergistic actions, they have shown distinct functional differences [39] and minimal common drive in isometric heel raises [40], which is hypothesized to directly correlate with muscle force production [41]. Although a small effect, some EMG information in the GA may have been contaminated with synaptic inputs unrelated to torque output in the intact limb task. Similarly, for residual muscles, we attempted to minimize crosstalk in our sensor placement, but following conventional surgeries, antagonist muscles can be closer in proximity with greater proportions of local subcutaneous adipose tissue compared to intact muscle [42]. Together, there is a higher probability of crosstalk occurring compared to intact muscles [43], [44]. Since AMP frequently cannot activate antagonist muscles fully independently and have greater variation in muscle activity [45], it is possible the relation of the macro-EMG information to the same torque output was inconsistent, leading to drift in steady-state torque predictions (Figure 4B). By decoding at the MU-level rather than the EMG-level, and through combination with a biomechanical model, we were able to recover and smooth the signal. By also refining MUs, we selected those with common drive most related and consistent with reference torque output.

One key contribution of this study was examining the feasibility of using a MU-based decoding neural interface to estimate torque of a missing joint for lower-limb prosthesis control. Although MU-based decoders have shown feasibility in residual muscles of AMP in the upper-limb for prosthesis control [17], [20], lower-limb muscles tend to have distinct differences in MU properties such as higher innervation numbers than lower-limb muscles, in addition to MU recruitment-coding (compared to rate-coding) of muscle activation [23], which may affect decoder performance. In addition, amputation surgeries have distinct strategies between upper- and lower-limbs. In the upper-limb, a large number of muscles in a complex anatomical region are affected. In contrast, lower-limb amputations affect fewer muscles, which are often displaced to provide cushion in weight-bearing for socket fittings [46]. With much larger affected muscles, surgical technique in the lower-limb may have a more asymmetric influence on the MU pool within a given muscle (since MU territories tend to be compartmentalized [47]), yielding different effects to EMG and decoders compared to EMG in upper-limbs. Furthermore, for upper-limb prosthesis control, most studies compared decoding methods in predicting kinematics [17], [20] since upper-limb prosthesis control is typically velocity- or position-based. Instead, for lower-limb prostheses, impedance control (requiring low-level torque control) [48] or torque control [49] are usually used to ensure user safety when interacting with different environments in walking or standing. All these differences motivated us to examine the feasibility of MU-based decoders applied to residual antagonistic ankle muscles for ankle torque estimation. Our study results showed a MU-based decoder is not only feasible but may be even more accurate than EMG-based methods. Hence, these findings contribute important knowledge for future development of a robust neural control interface for powered prosthesis ankles.

### B. Sensitivity Analysis & Model Refinement

Our Monte Carlo simulations revealed that torque predictions were most sensitive to the twitch force parameters *T_r_min__* and *T_hr_min__* for all muscles in the MUDrive model. With the design of the model, the minimum and maximum parameters were related based on the recruitment threshold for a given MU, with earlier recruited MUs having lower amplitudes, rise-times, and half-relaxation times [33], according to Henneman’s size principle [34]. Orderly recruitment coupled with the submaximal activations studied here likely biased activated MUs to RTs below 20-35 %MVC. The skewed distribution to lower RTs is a likely explanation for the observed sensitivity for the minimum parameter values compared to the maximum values (*T_r_max__* and *T_hr_max__*). Additionally, the gain factor applied to each twitch was based on both instantaneous firing rate and rise time. Hence, *T_r_* directly impacted rise time and amplitude, which may explain why it was the most sensitive parameter.

Because some parameters displayed little sensitivity to prediction performance, we successively reduced the number of parameters optimized to explore potential for reducing optimization time without sacrificing model performance. With fewer parameters optimized, optimization time reduced as expected (Figure 7, exceptions may have been due to the stochastic nature of the genetic algorithm to find a minimum in the cost function). The decrease in optimization time was smaller for the TA, but for both the residual and intact GA, a 6-7 sec reduction in optimization time occurred, corresponding to more than ten percent decrease for both cases. It is important to note the training data used for optimization included approximately 30 sec of data with one trapezoid of activation. In longer training sets with varied activations for more robust testing, the amount of time saved with model refinement may be substantially larger and relieve training time for calibration in a clinical training environment. Surprisingly, the default MUDrive model without any optimization of twitch parameters outperformed the aEMG and ND methods. This result differs from a similar earlier study in thumb abduction, where default parameterization of a twitch model was comparable to aEMG, but supervised regression learning to optimize the model significantly improved performance [21]. In that study, no MU refinement was conducted, and all decomposed MUs were input into the default twitch model, subjecting outcomes to potential errors in the decomposition or MUs with more variation in firings. Since only a few MUs may be needed to represent the common drive [50], refining the model to the most accurate and robust MUs may allow avoidance of extra complexity in optimizing the twitch model, if quick application of a MU-based controller was desired.

While optimizing fewer parameters still resulted in better outcomes compared to conventional methods, there was a trade-off with overall performance. Full optimization was needed to attain the best performance in the residual limb task (Table II), which exhibited more variation in muscle activations compared to the intact limb task. If better controller accuracy was desired to account for variations of muscle activity in more realistic scenarios, optimizing only *T_r_min__* as the most influential parameter could improve performance while maintaining faster optimization compared to optimizing all parameters. Because parameter sensitivity ranks were consistent, if performance was prioritized over optimization computation time, choosing parameters to successively include in optimization to improve performance would be trivial.

### C. Limitations & Future Work

This study has several limitations. First, as an initial investigation of MU-based torque estimation in (residual) shank muscles, we did not consider the coordination of the antagonistic muscles to compute net torque at the ankle joint. A more complex experimental protocol, such as balancing an inverted pendulum Fleming2019, would permit an agonist-antagonist model often used in direct EMG control. Second, analyses were conducted offline. We did so as a first step to minimize confounding factors for decoding with real-time hardware and demonstrate feasibility of MU-based decoding of intact and residual lower-limb muscle activity, as offline decoder performance is indicative of real-time performance [51]. Real-time MU decomposition is feasible [17], [18], so in the near-term, online control in a virtual environment needs to be explored. In the long-term, as integration of electrodes with lower-limb prosthesis sockets [52] and decomposing MUs in isotonic and dynamic activations [53], [54] improve, performance comparison in functional tasks with MU- versus EMG-based control is needed. Models were trained and tested in the same trial to maintain the active decomposed MU pool. Investigation of MU-based ankle torque estimation in less controlled scenarios across sessions and other evaluative metrics (e.g. waveform stability) are necessary to implement in settings outside the lab. Additionally, the mirrored task may have augmented error from actual motor intent, but such error would be constant across methods studied. Mirroring studies in the upper limb [27] need lower limb testing to understand the extent of error in this use case. Lastly, larger sampling of the population heterogeneity is necessary to illuminate relation of subject-specific factors such as muscle architecture or function level with model performance.

## V. Conclusion

Motivated by increasing research in myoelectric control of lower-limb prostheses, we investigated whether using MUs rather than EMG amplitude, the conventional input for direct control, would improve accuracy of motor intent decoding of lower-limb ankle joint torque. This work revealed that MU firings accurately predict isometric ankle dorsi- and plantarflexion torque using EMG signals from both intact and amputated muscle below the knee. Use of MU firings in a physiologic informed biomechanical model of muscle activation (MUDrive) significantly outperformed EMG amplitude methods in all cases. Additionally, we showed optimization of MUDrive parameters can be refined to speed up computational time and still maintain better outcomes to conventional EMG. Future applications of MU-based models may provide a more accurate control input signal to powered myoelectric prostheses and neural-machine interfaces and improve functional performance in postural or locomotive tasks.

## Supplementary Material

supp1-3336543

## Figures and Tables

**TABLE I:** Characteristics of People with Amputation

ID	Sex	Age	Height (cm)	Mass (kg)	Years Since Amputation	Cause
AMP1	M	53	174	103	6	Trauma
AMP2	M	19	180	83.3	4	Trauma
^†^AMP3	M	52	196	119	3	Intractable Pain
AMP4	M	58	177	81.1	1	*PAD
AMP5	M	42	183	99.3	19	Trauma
AMP6	F	67	165	74.4	9	*PAD
AMP7	M	30	175	122	19	Trauma
Mean (±SD)	--	45.6 (16.7)	179 (9.54)	97.4 (18.7)	8.71 (7.45)	--

* Peripheral Arterial Disease,

† Partial foot amputation on contralateral limb

**TABLE II:** Change in Performance (RMSE) with Model Refinement

	TA						GA					

Parameters Optimized	0	1	2	3	4	5 (Full)	0	1	2	3	4	5 (Full)
Residual	**2.095 (0.204)**	**2.060 (0.204)**	**2.032 (0.202)**	**2.009 (0.199)**	2.004 (0.199)	2.004 (0.199)	**4.447 (0.349)**	**4.295 (0.343)**	**4.217 (0.345)**	**4.093 (0.331)**	**4.069 (0.326)**	4.060 (0.326)
Intact	**0.862 (0.049)**	**0.842 (0.048)**	0.818 (0.047)	0.808 (0.047)	0.809 (0.048)	0.808 (0.048)	**2.913 (0.200)**	**2.868 (0.191)**	**2.822 (0.190)**	2.771 (0.190)	2.751 (0.185)	2.749 (0.185)
NON	**1.051 (0.043)**	**1.003 (0.042)**	**0.986 (0.042)**	0.953 (0.039)	0.952 (0.039)	0.951 (0.039)	**3.148 (0.129)**	**3.072 (0.128)**	**3.001 (0.125)**	2.931 (0.124)	2.934 (0.124)	2.927 (0.124)

Bold values indicate significantly worse performance compared to a fully optimized model (paired t-tests).
