# Supplementary material for: Ankle Torque Estimation With Motor Unit Discharges in Residual Muscles Following Lower-Limb Amputation
Source: IEEE Trans Neural Syst Rehabil Eng. Author manuscript; Available in PMC 2023 Dec 27. (PMC10752569; doi:10.1109/TNSRE.2023.3336543)
Supplement: supp1-3336543 [file NIHMS1950691-supplement-supp1-3336543.pdf]

## SUPPLEMENTARY MATERIAL

*MUDrive Model Training Details*

The same refined MUs used in the ND method were kept for the MUDrive method. The MUDrive method is derived from a simulation model that estimates a modeled force twitch profile for a given MU [1]. The twitch profiles of refined MUs were convolved with their respective decomposed spike trains and summed to compute a MUDrive signal over time, representing modeled force (Figure 3C). This method has been implemented in decoding motor intent for AMP in the upper-limb [2]. The twitch force $f$ for the $i$th MU is represented by a function of time $t$ by Eqn. 2:

$$f_i(t) = pt^m e^{-kt} \qquad (2)$$

where $p, m,$ and $k$ are constants. The amplitude $p$ is defined as

$$p = P_i \left(\frac{e}{T_{r,i}}\right)^{kT_{r,i}} \qquad (3)$$

where $P_i$ and $T_{r,i}$ are the twitch force peak amplitude and rise time to peak amplitude, respectively. The decay rate $k$ is defined as

$$k = \frac{\ln(2)}{T_{hr,i} - T_{r,i}\ln(1+\frac{T_{hr,i}}{T_{r,i}})} \qquad (4)$$

where $T_{hr,i}$ is the half-relaxation time (time from $P_i$ to $P_i/2$), and $m$ is dependent on the decay rate and rise time,

$$m = kT_{r,i} \qquad (5)$$

A gain factor $g_{ij}$ for each MU at the $j$th firing was implemented in accordance with representing isolated twitches up to tetanus force production.

$$g_{ij}(fn_{ij}) = \begin{cases} 1, \; 0 < fn_{ij} \leq 0.4 \\ \frac{0.4}{fn_{ij}(1-r)}[1 - re^{\frac{0.4-fn_{ij}}{c}}], \; fn_{ij} > 0.4 \end{cases} \quad (6)$$

$$fn_{ij} = \frac{T_{r,i}}{IPI_{ij}} \quad (7)$$

where $c$ and $r$ are constants (0.85 and 2.13, respectively [1], [2]), and $fn_{ij}$ is a function of the rise time and inter-pulse interval $IPI_{ij}$, i.e., time since the prior firing of the MU.

Larger MUs tend to have longer rise times and half-relaxation times and larger peak force [3]. These three parameters for a given MU were computed as a linear rescaling to the range of the parameter for the MU pool based on its recruitment threshold (RT), according to the size principle [4]. The RT for each MU was defined as the %MVC of the EMG signal amplitude in a 200 ms window centered at the time of the first firing when the MU firing rate was greater than 5 Hz. As an example, to compute the rise time of the $i$th MU,

$$T_{r,i} = \frac{RT_i \,\%MVC}{100 \,\%MVC}\left(T_{r\_max} - T_{r\_min}\right) + T_{r\_min} \quad (8)$$

The same moving window and Kalman filter were applied to the MUDrive signal, and the smoothed output was linearly regressed to the recorded torque. As done in [2], a genetic algorithm optimized MUDrive model performance in each trial to determine the maximum and minimum peak twitch amplitude, rise times, and half-relaxation times. In this study, based on [1], the constrained ranges to determine $P_{max}$, $T_{r\_min}$, $T_{r\_max}$, $T_{hr\_min}$, and $T_{hr\_max}$ were 1.1-150 (arb. unit), 30-110 ms, 150-240 ms, 15-60 ms, and 110-140 ms, respectively (with $P_{min}$ predefined at 1).

*Statistical Model Results*

Table S1 displays F-Statistics for fixed effects from an analysis of variance on the linear mixed effects model for each condition. In all cases significant differences were observed for both Method and %MVC level. No significant interactions were observed between these effects, so results across %MVC levels were combined to summarize data for each method as the primary research question in the main manuscript. Table S2 displays the partial-eta-squared for each effect in each condition. Commonly, values of 0.01, 0.06, and 0.14 are used as thresholds for small, medium, and large effects, respectively [5]. Both Method and %MVC Level had a medium or large effect on RMSE in all conditions except %MVC Level in the intact TA. Only small interaction effects were observed in all conditions. Details on model fitted coefficients for each fixed effect can be found in Table S3.

Table S1: F-Statistics for Fixed Effects

| | TA | | | GA | | |
|---|---|---|---|---|---|---|
| Effect | Residual | Intact | NON | Residual | Intact | NON |
| Method | **5.16** | **6.34** | **12.3** | **24.3** | **8.47** | **17.4** |
| %MVC Level | **16.0** | **4.71** | **6.94** | **33.7** | **9.91** | **19.4** |
| %MVC Level:Method | 0.791 | 2.59 | 2.35 | 0.386 | 0.476 | 2.99 |

**Bold** values indicate statistically significant differences.
The critical F-stat was 3.11.

Table S2: Partial-Eta-Squared for Fixed Effects

| | TA | | | GA | | |
|---|---|---|---|---|---|---|
| Effect | Residual | Intact | NON | Residual | Intact | NON |
| Method | 0.118 | 0.126 | 0.117 | 0.37 | 0.161 | 0.158 |
| %MVC Level | 0.172 | 0.051 | 0.272 | 0.291 | 0.184 | 0.095 |
| %MVC Level:Method | 0.020 | 0.055 | 0.025 | 0.009 | 0.011 | 0.031 |

Table S3: Fixed Effect Coefficients

| Effect | TA Residual | | TA Intact | | TA NON | |
|---|---|---|---|---|---|---|
| (Intercept) | 2.26 | (0.57) | 0.835 | (0.18) | 1.11 | (0.098) |
| aEMG | 0.474 | (0.27) | 0.374 | (0.12) | 0.339 | (0.083) |
| ND | 0.710 | (0.27) | 0.266 | (0.12) | 0.273 | (0.083) |
| 20 %MVC | -0.519 | (0.27) | -0.056 | (0.12) | -0.316 | (0.083) |
| 20 %MVC:aEMG | 0.0801 | (0.38) | -0.310 | (0.17) | -0.224 | (0.12) |
| 20 %MVC:ND | -0.363 | (0.38) | 0.0344 | (0.17) | -0.0115 | (0.12) |

| Effect | GA Residual | | GA Intact | | GA NON | |
|---|---|---|---|---|---|---|
| (Intercept) | 4.50 | (0.97) | 3.06 | (0.55) | 3.07 | (0.42) |
| aEMG | 2.31 | (0.47) | 0.709 | (0.33) | 1.59 | (0.28) |
| ND | 1.70 | (0.47) | 1.13 | (0.33) | 0.948 | (0.28) |
| 20 %MVC | -1.24 | (0.47) | -0.612 | (0.33) | -0.286 | (0.28) |
| 20 %MVC:aEMG | -0.332 | (0.65) | -0.196 | (0.47) | -0.938 | (0.40) |
| 20 %MVC:ND | -0.547 | (0.65) | -0.441 | (0.47) | -0.312 | (0.40) |

The mean ($\pm$ standard error) for each fitted coefficient is displayed.

*MVC Fixed Effect Results*

In all cases the targeted 35 %MVC level had a significantly higher error than the lower 20 %MVC level (Figure S1). Maintaining 35 %MVC steady-state activation levels tended to result in more varied changes in muscle activation relative to changes in torque compared to the 20% MVC condition. Higher variability in muscle activation while maintaining a similar level of torque output may have contributed to larger errors observed. Studies examining torque estimation across a larger range of profile types and %MVC levels are needed to determine if this effect is consistent beyond the 20-35 %MVC range studied.

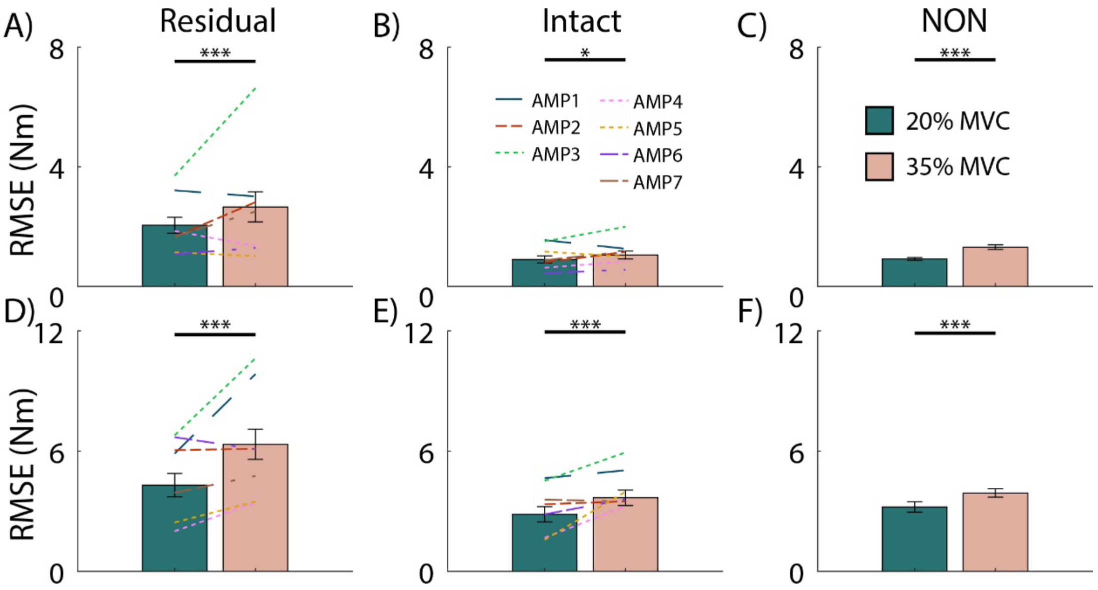


Figure S1: Summary of performance across methods at each MVC level in each muscle. Panels in the top row show data from the TA of the A) residual limb, B) intact limb, and C) limbs of NON. The bottom row (D-F) shows data from the GA muscles for the respective limbs corresponding to the top row. Each bar displays the mean and standard error for each MVC level across all trials in all. Line plots show averages for each subject (*p<0.05, **p<0.01, ***p<0.001).

## REFERENCES


[1] P. Contessa and C. J. De Luca, “Neural control of muscle force: Indications from a simulation model,” *Journal of Neurophysiology*, vol. 109, no. 6, pp. 1548–1570, 2013, doi: 10.1152/jn.00237.2012.

[2] M. D. Twardowski, S. H. Roy, Z. Li, P. Contessa, G. De Luca, and J. C. Kline, “Motor unit drive: A neural interface for real-time upper limb prosthetic control.,” *Journal of Neural Engineering*, vol. 16, no. 1, p. 016012, Feb. 2019, doi: 10.1088/1741-2552/aaeb0f.

[3] S. Andreassen and L. Arendt-Nielsen, “Muscle fibre conduction velocity in motor units of the human anterior tibial muscle: a new size principle parameter.,” *The Journal of Physiology*, vol. 391, no. 1, pp. 561–571, 1987.

[4] E. Henneman, “Relation between Size of Neurons and Their Susceptibility to Discharge,” *Science*, vol. 126, no. 3287, p. 1345 LP – 1347, Dec. 1957, doi: 10.1126/science.126.3287.1345.

[5] J. Cohen, *Statistical power analysis for the behavioral sciences*. Academic press, 2013.
